# Toosendanin Exerts an Anti-Cancer Effect in Glioblastoma by Inducing Estrogen Receptor β- and p53-Mediated Apoptosis

**DOI:** 10.3390/ijms17111928

**Published:** 2016-11-18

**Authors:** Liang Cao, Dingding Qu, Huan Wang, Sha Zhang, Chenming Jia, Zixuan Shi, Zongren Wang, Jian Zhang, Jing Ma

**Affiliations:** 1 Department of Traditional Chinese Medicine, Xijing Hospital, Fourth Military Medical University, Xi’an 710032, China; caoliang402@fmmu.edu.cn (L.C.); zhangsha@fmmu.edu.cn (S.Z.); jiacm2014@sina.com (C.J.); zongren@fmmu.edu.cn (Z.W.); 2 Department of Biochemistry and Molecular Biology, Fourth Military Medical University, Xi’an 710032, China; qudingd@hotmail.com (D.Q.); pfkwhuan@163.com (H.W.); 3 Department of Acupuncture, Shaanxi Hospital of Traditional Chinese Medicine, Xi’an 710032, China; stone_x0319@163.com

**Keywords:** glioblastoma, toosendanin, apoptosis, estrogen receptor β, p53

## Abstract

Glioblastoma (GBM) is the most common primary brain tumor with median survival of approximately one year. This dismal poor prognosis is due to resistance to currently available chemotherapeutics; therefore, new cytotoxic agents are urgently needed. In the present study, we reported the cytotoxicity of toosendanin (TSN) in the GBM U87 and C6 cell lines in vitro and in vivo. By using the MTT (3-(4,5-dimethyl-2-thiazolyl)-2,5-diphenyl-2-*H*-tetrazolium bromide) assay, flow cytometry analysis, and Western blot, we found that TSN inhibited U87 and C6 cell proliferation and induced apoptosis at a concentration as low as 10 nM. Administration of TSN also reduced tumor burden in a xenograft model of athymic nude mice. Pharmacological and molecular studies suggested that estrogen receptor β (ERβ) and p53 were prominent targets for TSN. GBM cell apoptosis induced by TSN was a stepwise biological event involving the upregulation of ERβ and contextual activation of functional p53. Collectively, our study indicates, for the first time, that TSN is a candidate of novel anti-cancer drugs for GBM. Furthermore, ERβ and p53 could act as predictive biomarkers for the sensitivity of cancer to TSN.

## 1. Introduction

Glioblastoma (GBM) is the most frequent and life-threatening primary malignancy in the central nervous system. Surgical resection followed by either radiation therapy or adjuvant chemotherapy remains the gold standard for GBM. However, despite advances in treatment strategies, the prognosis of patients with GBM remains unfavorable, with a two-year survival rate of less than 5% [1]. Although recent understandings regarding the molecular mechanisms of GBM tumorigenesis and progression have largely redefined its classification and potentially identified new treatment approaches, the clinical outcome of patients has not yet improved [2,3,4]. Therefore, it is urgent to discover effective therapeutic targets and agents to provide clinical benefits for GBM patients.

The estrogen receptor (ER) is found in various tissues and organs and acts as a hormone receptor for sex steroids. The canonical ER pathway is activated by estrogen, in which ER bound to its ligand rapidly to translocate into the nucleus and forms ER-ER dimers to bind to specific DNA sequences known as hormone response elements [5,6]. The ER/DNA complex subsequently recruits transcription coactivators such as AP-1, SP-1, and NF-κB to activate the expression of ER-targeted genes [7,8]. Meanwhile, ER also acts as an adaptor protein and exerts additional functions independent of DNA binding [9]. For instance, ER is usually associated with membrane receptor tyrosine kinases to send survival signals to the nucleus through the phosphatidylinositol 3 kinase (PI3K)/Akt pathway. Phosphorylation of ER by receptor tyrosine kinases increases its transcriptional activity, whereas dephosphorylation of ER suppresses this effect [10]. ER also interacts with BRCA1 to regulate the production of vascular endothelial growth factor in breast cancer [11]. To date, two major forms of the *ER* gene have been identified. The *ERα* gene (also known as *ESR1*) is located on chromosome 6q25.1 and encodes ERα protein, and its homologous counterpart *ERβ* (also known as *ESR2*) is located on chromosome 14q23.2. Although these two ERs share 97% homology in their DNA binding domains, they exhibit contradictory biological functions. *ERα* is generally believed to be an oncogene and promotes cell proliferation, whereas *ERβ* is anti-proliferative and acts as a putative tumor suppressor [12,13,14]. A genome-wide study has indicated that ERα and ERβ proteins differentially regulate genes involved in cell growth and survival. This unique molecular property of ERs highlights a therapeutic strategy that downregulating ERα and upregulating ERβ may be beneficial in treating cancers that express ERs [15,16,17]. Indeed, an ERα antagonist-based endocrine therapy for ERα-positive breast cancer has been fairly well characterized and has largely improved patient prognosis and prolonged patient survival. Moreover, ERβ-specific agonists have been implicated to exert benefits in ERβ-deficient advanced colon cancer [18,19]. GBM expresses both ERα and ERβ, but the significance of targeting the specific ERs remains to be elucidated. ER ligands as potential therapy for GBM are also limited due to their adverse effect in the reproductive system and cardiovascular system. Thus, selective ER agonists derived from natural compounds are currently being investigated for potential therapeutic applications.

Toosendanin (TSN), a triterpenoid saponin extracted from the medicinal herb *Melia toosendan* Sieb. et Zucc., has been used to kill parasites and agricultural insects in East Asia for more than 2000 years. Since the identification of the chemical structure of TSN in the 1980s, a large number of studies have been conducted to investigate its biological effects. It has been reported that TSN selectively blocked acetylcholine release from nerve terminals and revealed anti-botulismic activity [20,21]. Moreover, TSN possesses anti-proliferative and apoptosis-inducing effects on various human cancer cells in vitro, including hepatocellular carcinoma, leukemia, and lymphoma. The underlying molecular mechanism that has been implicated involves the suppression of the PI3K/Akt, MEK/Erk and Mitogen-activated protein kinase (MAPK)/c-Jun N-terminal kinase (JNK) pathways [22,23]. However, the effect of TSN on GBM cell proliferation and apoptosis has not been elucidated.

In the present study, we evaluated the cytotoxicity of TSN in GBM cells in vitro and in vivo. Our results suggested that TSN inhibits the proliferation of distinct subtypes of GBM cells and induces apoptosis. Intriguingly, we identified a specific association between ERβ and the p53 status in response to TSN treatment in GBM. TSN treatment leads to apoptosis in ERβ-positive GBM cells through contextual upregulation of p53. It is therefore essential to determine the patient’s ERβ and p53 status before treating GBM with a TSN-based strategy.

## 2. Results

### 2.1. TSN (Toosendanin) Inhibits GBM (Glioblastoma) U87 and C6 Cell Proliferation

**Figure 1:**
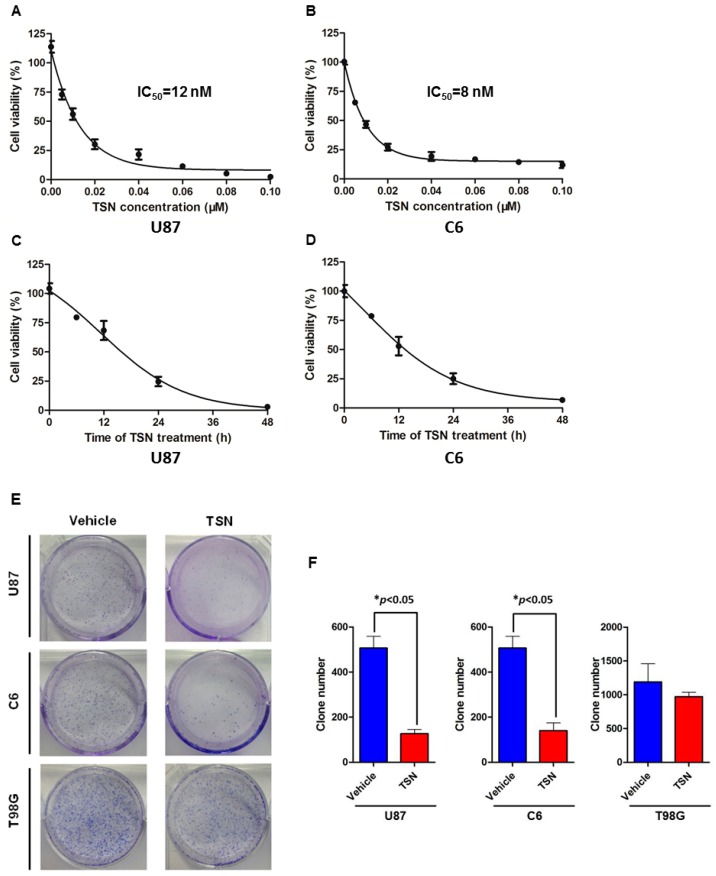
Toosendanin (TSN) inhibited glioblastoma (GBM) cell proliferation. (**A**) U87 cells were treated with increasing concentrations (5–100 nM) of TSN for 48 h and assessed for growth inhibition by the MTT (3-(4,5-dimethyl-2-thiazolyl)-2,5-diphenyl-2-*H*-tetrazolium bromide) assay; (**B**) The effect of TSN on C6 cell proliferation was assessed as described in (**A**); (**C**) U87 cells were treated with 10 nM TSN for different time intervals (0, 6, 12, 24, and 48 h) and assessed for cell proliferation; (**D**) C6 cells were treated and assessed as described in (**C**); (**E**) Representative images of GBM cell colony formation. GBM cells were seeded in a six-well plate and treated with either 1 nM TSN or an equal volume of vehicle for 10 days. Cell colonies were stained with Giemsa and counted; (**F**) Statistical analysis of the colony numbers (* *p* < 0.05).

The growth inhibitory effect of TSN on GBM cells was assessed by the MTT (3-(4,5-dimethyl-2-thiazolyl)-2,5-diphenyl-2-*H*-tetrazolium bromide) assay. The results showed that TSN inhibited the growth of human GBM U87 cells in a dose- and time-dependent manner (Figure 1,C). Moreover, TSN also showed inhibitory activity on the growth of rat GBM C6 cells. As shown in Figure 1B,D, TSN inhibited C6 cell growth in a dose- and time-dependent manner. The IC_50_ values at 48 h in U87 and C6 cells were 12 and 8 nM, respectively. Therefore, TSN at a final concentration of 10 nM was used in all subsequent experiments unless otherwise specified.

We next evaluated the growth inhibitory effect of TSN on U87 and C6 cells over an extended time interval. U87 and C6 cells were seeded in a six-well plate and treated with 1 nM TSN for 10 days. At the end of the experiment, the colonies were fixed and visualized by Giemsa staining. Statistical analysis indicated that TSN treatment resulted in a significant reduction in the number of colonies formed by the U87 and C6 cells compared with the vehicle-treated cells (Figure 1E,F). These results suggested that TSN is cytotoxic to GBM U87 and C6 cells and inhibits U87 and C6 cell proliferation over both short and long time intervals.

### 2.2. TSN Induces GBM U87 and C6 Cell Apoptosis In Vitro

**Figure 2:**
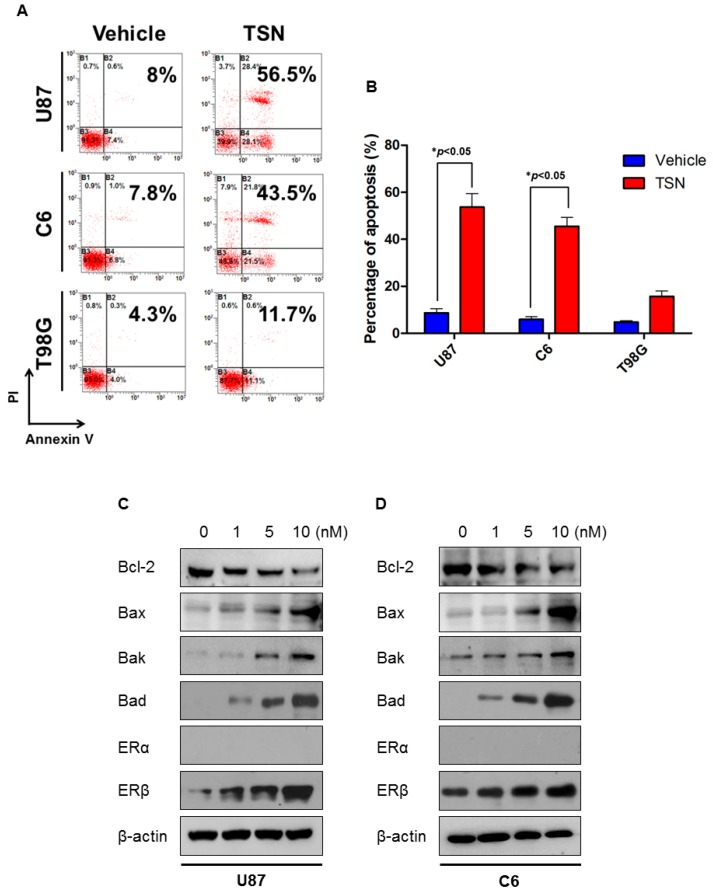
Effect of TSN on GBM cell apoptosis. (**A**) Representative images of GBM cell apoptosis after TSN treatment. GBM cells were treated with either 10 nM TSN or an equal volume of vehicle for 48 h and evaluated for apoptosis by Annexin V/PI double staining; (**B**) Statistical analysis of the percentage of apoptotic GBM cells (* *p* < 0.05); (**C**,**D**) Western blot analysis of apoptosis-related proteins and estrogen receptor (ER) proteins in TSN-treated U87 and C6 cells.

To investigate whether the cytotoxic effect of TSN was related to the induction of apoptosis, flow cytometry was performed after the indicated treatment. In agreement with the MTT assay, TSN treatment resulted in apoptosis of U87 and C6 cells. As illustrated in Figure 2A,B, nearly 50% of the cells underwent either early stage or late stage apoptosis in response to TSN, which is significantly different from the cells treated with vehicle. The percentage of Annexin V−/PI+ cells, which represented cells undergoing necrosis, did not change significantly after TSN treatment, suggesting that TSN prominently promoted U87 and C6 cell apoptosis but not necrosis.

Western blot analysis of apoptosis-related proteins further confirmed the induction of apoptosis by TSN. Figure 2C shows that TSN dose-dependently decreased the expression of the anti-apoptotic protein Bcl-2 and increased the expression of the pro-apoptotic proteins Bax, Bak, and Bad in U87 cells. Similarly, in C6 cells, the Bcl-2 protein content was dramatically reduced, whereas the Bax, Bak, and Bad protein content was increased in the presence of TSN (Figure 2D). Among the three tested doses, 10 nM TSN led to the most evident changes in apoptosis-related proteins in both cell lines.

### 2.3. TSN Induces GBM U87 Cell Apoptosis In Vivo

**Figure 3:**
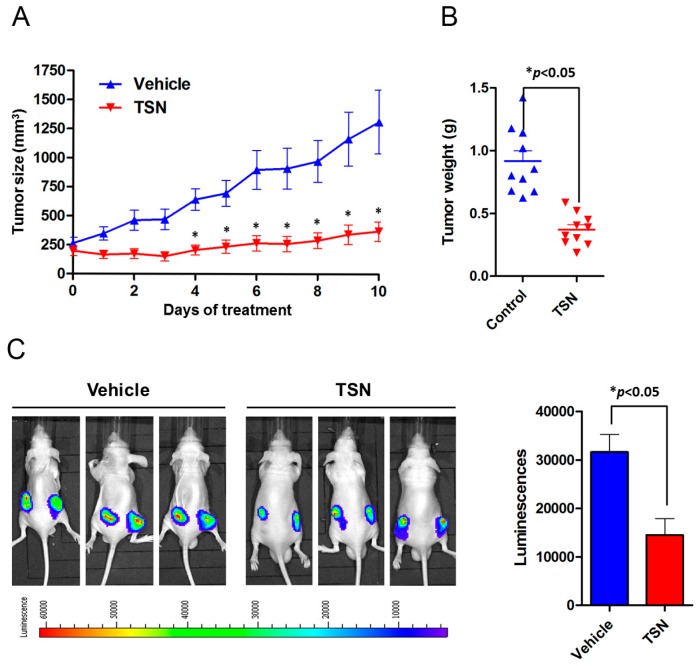

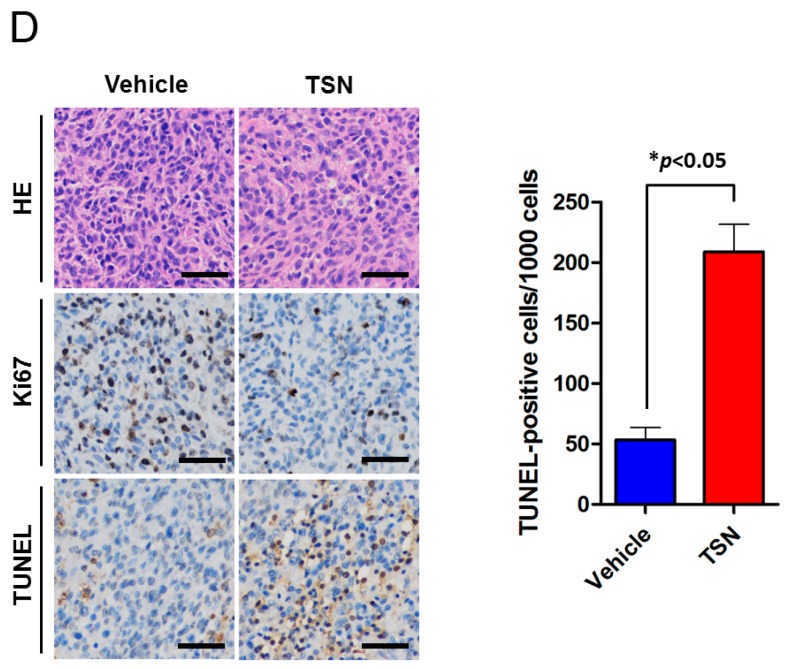
Anti-cancer effect of TSN on U87 cells in vivo. (**A**,**B**) U87-Luc cells were subcutaneously implanted into six-week-old athymic nude mice, and the mice were treated with either TSN or vehicle. The volume of the xenograft tumors was measured daily. After 10 days of treatment, the tumor nodules were isolated and weighted; (**C**) Measurement of the bioluminescence signal by intraperitoneal injection of D-Luciferin. Representative images and statistical analysis of the luminescence are illustrated (* *p* < 0.05); (**D**) Histological analysis of U87 cell proliferation and apoptosis by Ki67 staining and terminal deoxynucleotidyl transferase-mediated dUTP nick end labeling (TUNEL) assay (* *p* < 0.05). Scale bar indicates 100 µm.

To evaluate the cytotoxicity of TSN on in vivo tumor growth and apoptosis, U87 cells stably expressing luciferase (U87-Luc) were subcutaneously injected into the bilateral flanks of six-week-old athymic nude mice. Once the xenograft reached a suitable size, the mice were randomized into a control group, which received vehicle, and a treatment group, which received TSN (1 mg/kg qd) via oral gavage. The tumor size was monitored by vernier calipers daily. The U87 tumor size was dramatically increased in the vehicle group. At the end of the experiment, the tumor burden achieved a five-fold increase compared to the baseline. In contrast, TSN treatment significantly reduced tumor progression in the U87 xenograft model (Figure 3A). Consistently, the weight of the tumor nodules in the TSN group was much lower than that in the vehicle group (Figure 3B). Measurement of luciferase intensity also revealed that TSN treatment led to a sharp reduction in the bioluminescence signal (Figure 3C). Immunohistochemistry staining of the tumor nodules demonstrated that TSN markedly reduced the expression of the proliferative marker Ki67 and increased the number of terminal deoxynucleotidyl transferase-mediated dUTP nick end labeling (TUNEL)-positive apoptotic cells (Figure 3D). To further confirm apoptosis induction by TSN in vivo, the expression levels of Bcl-2, Bax, and cleaved caspase-3 were examined. As shown in Figure S1, TSN treatment increased the expression of Bax and cleaved caspase-3 while reducing Bcl-2 expression compared to the vehicle group. Collectively, these results suggested the in vivo cytotoxic activity of TSN on GBM U87 cells and that TSN inhibited U87 xenograft progression by inducing apoptosis.

### 2.4. TSN Does Not Exhibit Cytotoxicity in GBM T98G Cells

Having established the cytotoxic and apoptosis-inducing effect of TSN on GBM U87 and C6 cells, we next examined the activity of TSN in another widely used GBM cell line, T98G. To our surprise, T98G cells exhibited the opposite response to TSN. U87 and C6 cells were extremely sensitive to TSN treatment, which had nanomolar activity on cell proliferation and apoptosis. However, T98G cells were more resistant to this activity with an IC_50_ value over 0.2 μM, which was nearly 20-fold higher than that of U87 and C6 cells (data not shown). The T98G colonies continued to grow in the presence of 1 nM TSN, and the number of colonies in TSN group was comparable to that in the vehicle group (Figure 1E,F). Induction of apoptosis by TSN was also compromised in T98G cells. Although there was a slight increase in the percentage of apoptotic cells after 10 nM TSN treatment, this alteration was not evident (Figure 2A,B). TSN at 1–10 nM also failed to affect the expression of Bcl-2 family proteins in T98G cells (Figure S2). This apparent discrepancy led us to consider the genetic difference of the three tested cell lines and investigate the underlying mechanism of apoptosis induced by TSN.

### 2.5. ERβ (Estrogen Receptor β) Is Required for the Cytotoxicity of TSN

**Figure 4:**
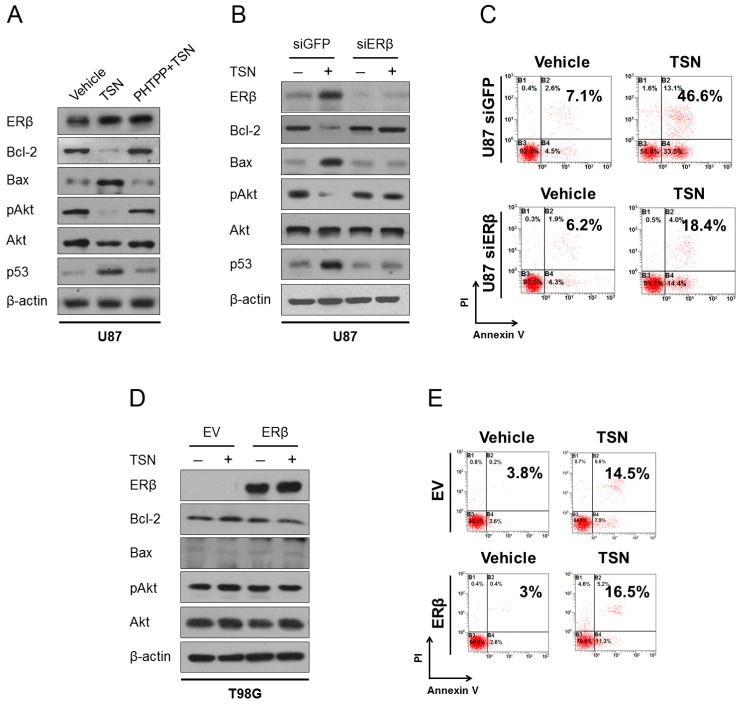
Estrogen receptor β (ERβ) is required for apoptosis induction by TSN. (**A**) U87 cells were treated with 10 nM TSN either with or without the selective ERβ antagonist PHTPP for 48 h and evaluated for Bcl-2, Bax, phosphorylated Akt, and p53 expression; (**B**) U87 cells were transfected with a specific siRNA against ERβ and treated with 10 nM TSN for an additional 48 h. The expression of Bcl-2, Bax, pAkt, and p53 were analyzed by Western blot. siRNA against GFP was used as a control for the transfection procedure; (**C**) Flow cytometry analysis of apoptosis in U87 cells transfected with siERβ either with or without TSN treatment. siRNA targeting GFP was used as a control; (**D**) ERβ-negative T98G cells were transfected with either an ERβ-expressing plasmid or empty vector (EV), treated with 10 nM TSN, and evaluated for Bcl-2, Bax, and pAkt protein levels; (**E**) The T98G/EV and T98G/ERβ cells were treated with either TSN or vehicle for 48 h and evaluated for apoptosis by flow cytometry.

Estrogens have been implicated in the development and differentiation of the central nervous system, and expression of ERα and ERβ overlap in distinct regions of the brain. Of special interest, ERβ acts as one of the tumor suppressors in the brain, and its deficiency has been observed in high-grade glioma and is associated with poor clinical outcomes [24,25]. By analyzing the genetic background of U87, C6, and T98G cells, we noticed that the TSN-sensitive U87 and C6 cells selectively expressed endogenous ERβ, whereas the TSN-resistant T98G cells selectively expressed the α isoform of ER (Table 1). To investigate the effect of TSN on ERα and ERβ expression, cells were treated with TSN for 48 h and subjected to Western blot. TSN was found to dose-dependently increase ERβ protein levels in U87 and C6 cells, but it did not induce the expression of ERα. Meanwhile, in T98G cells—which uniquely express ERα—TSN did not change the ERα protein levels. TSN administration also upregulated ERβ protein level in U87 xenograft models. These results indicated that ERβ, but not ERα, is a prominent target for TSN (Figure 2C,D, Figures S1 and S2). To test whether the apoptotic response to TSN is associated with ERβ induction, we inhibited ERβ by using pharmacological and molecular approaches. PHTPP is a selective ERβ antagonist, and preconditioning cells with PHTPP blocked the changes in apoptosis-related proteins and Akt dephosphorylation in U87 cells (Figure 4A). Moreover, knockdown of endogenous ERβ by using a specific siRNA oligo also compromised the pro-apoptotic effect of TSN in U87 cells, while a control siRNA targeting GFP had no effect (Figures 4B,C, Figure S3A). It is therefore reasonable to believe that ERβ is an important therapeutic target for TSN in GBM and that its upregulation is required for the induction of apoptosis.

To further support our hypothesis, we stably overexpressed ERβ in T98G cells (referred as T98G/ERβ cells) and speculated that the T98G/ERβ cells would become sensitive to TSN treatment. However, Western blot and flow cytometry analysis suggested that ectopic expression of ERβ failed to restore the sensitivity to TSN despite the efficient overexpression of ERβ protein. In T98G/ERβ cells, the addition of TSN neither decreased Bcl-2 protein expression and Akt phosphorylation nor increased Bax protein expression (Figure 4D). Quantitative analysis by flow cytometry showed that ectopically overexpressed ERβ failed to increase apoptosis induction by TSN (Figure 4E, Figure S3B). These paradoxes implied that although ERβ is a prominent target and is essential for TSN-induced apoptosis in GBM cells, it may not be the direct apoptotic executor of this activity. Proteins that are targeted by ERβ or proteins that are able to interact with ERβ would thus be responsible for the direct induction of apoptosis.

### 2.6. p53 as the Apoptotic Executor of TSN

Finally, we sought to identify the exact apoptotic executor of TSN in GBM cells. A recent study in colon cancer indicated that overexpression of ERβ induces LoVo cell apoptosis by increasing p53 content without TNF-α involvement [26]. By re-checking the p53 status in GBM cells, it was exciting to discover that U87 and C6 cells express wild type p53 (Table 1). However, although T98G cells also express p53, a genomic M237I mutation within the *p53* allele inactivates its DNA binding activity and leads to its loss-of-function. The M237I mutant *p53* gene in T98G cells therefore encodes a protein product that does not preserve the tumor suppressive function of wild-type p53, which may be a rational explanation of the unresponsiveness of T98G/ERβ cells to TSN. It was speculated that TSN exhibited cytotoxicity to GBM cells through the following contextual mechanism: (1) involvement of ERβ and (2) the presence of functional p53. Indeed, TSN readily upregulated the expression of p53 in U87 and C6 cells. The absence of ERβ in T98G cells abrogated the p53 response to TSN, although the baseline level of p53 in this cell line was comparable to that in U87 and C6 cells (Figure S4). Another important finding is shown in Figure 4, in which ectopic expression of ERβ in T98G cells expressing the M237I-mutated p53 failed to restore the apoptotic response to TSN. These data also suggested that apoptosis induction by TSN in GBM cells is a stepwise biological event involving the upregulation of ERβ and functional p53 (Figure 4A,B, Figure S1). These two tumor suppressors worked synergetically to mediate the cytotoxicity of TSN.

**Figure 5:**
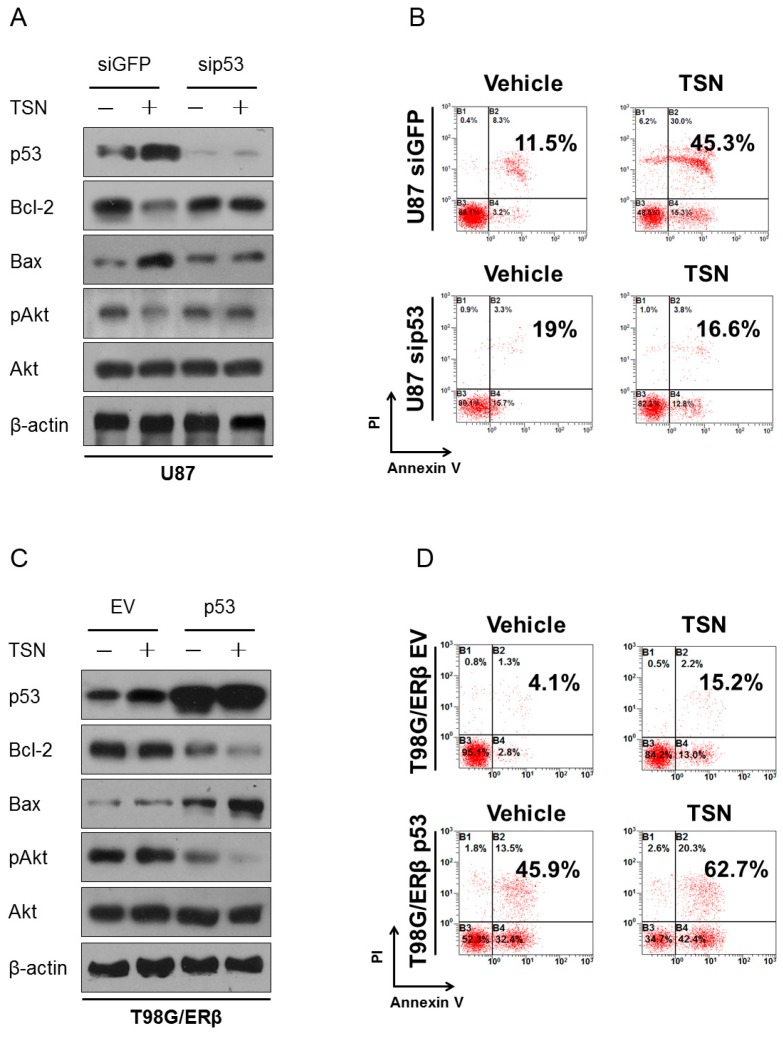
p53 as the apoptotic executor of TSN. (**A**,**B**) U87 cells were transfected with siRNA targeting p53 and treated with 10 nM TSN for an additional 48 h. The expression of Bcl-2, Bax, and pAkt and flow cytometry were evaluated to determine apoptosis. siRNA against GFP was used as a control for the transfection procedure; (**C**,**D**) The T98G/ERβ cells expressing M237I-mutated p53 were transfected with either wild-type p53 plasmid or empty vector (EV), treated with 10 nM TSN, and evaluated for apoptosis by Western blot and flow cytometry.

To investigate the exact role of p53 in the TSN response and in apoptosis induction, we knocked down endogenous p53 expression in U87 cells and overexpressed wild-type p53 (i.e., the functional form of p53) in T98G/ERβ cells. In agreement with Hsu and colleagues [26], silencing p53 abolished apoptosis and Akt dephosphorylation (Figure 5A,B, Figure S3C). In T98G cells stably expressing ERβ, TSN treatment increased the protein levels of p53. Since the endogenous p53 in T98G cells is not functional, as a consequence, Bcl-2, Bax, and pAkt protein expression is not affected by TSN treatment. In contrast, the T98G/ERβ cells transfected with wild-type p53 dramatically responded to TSN. Overexpression of wild-type p53 was sufficient to reduce Bcl-2 expression and Akt phosphorylation as well as increase Bax expression. The addition of TSN further enhanced the apoptotic response (Figure 5C,D, Figure S3D). Taken together, these findings indicated that upregulation of ERβ and p53, two major tumor suppressors in the central nervous system, are both required for the pro-apoptotic action of TSN in GBM. Selective overexpression of ERβ in p53-mutated GBM cells failed to restore TSN cytotoxicity (Figure 4D,E), whereas silencing wild-type p53 abrogated TSN-induced GBM cell apoptosis (Figure 5A,B), all of which strongly support a causal association between ERβ upregulation and p53 activity in response to TSN treatment.

## 3. Discussion

We reported the cytotoxic activity of TSN in GBM cells. Despite preclinical studies in breast cancer, hepatocellular carcinoma, leukemia, and prostate cancer that have shown the anti-cancer effect of TSN in various human malignancies, its clinical application in cancer treatment is limited because of liver injury when high concentrations of TSN were used in animal models. However, treating GBM with TSN is still promising due to the following: (1) TSN is able to cross the blood-brain barrier and exerts intracranial activity on neurotransmitter release and ion channels function [21]; (2) TSN elicits cytotoxicity to most cancer cells at nanomolar concentrations, which can be easily achieved by oral administration without causing obvious hepatotoxicity; and (3) GBM is usually resistant to chemotherapy, and new therapeutic options are warranted. These concerns have prompted us to investigate the pharmacological properties of TSN on GBM, and we are excited to report the in vitro and in vivo activities of TSN in U87 and C6 cells. TSN was found to inhibit U87 and C6 cell proliferation and colony formation as well as induce apoptosis. Meanwhile, administration of TSN at 1 mg/kg by oral gavage significantly reduced tumor burden in athymic nude mice. Of special interest, TSN at this dose is well tolerated without causing obvious hepatotoxicity and neurotoxicity. A recent study showed that liver toxicity occurred when mice were treated with 80 mg/kg TSN for nine days [27]. Thus, TSN may be a promising agent for GBM treatment and a combination with hepatoprotective/neuroprotective agents would lead to greater tolerance and clinical benefit to GBM patients.

However, when we applied this finding in the human GBM T98G cell line, we observed a contradictory response. T98G cells were tolerant to TSN at concentrations that were sufficient to cause growth inhibition and extensive apoptosis in U87 and C6 cells. These discrepancies suggested that TSN is cytotoxic to distinct subtypes of GBM, and identification of biomarkers predicting sensitivity to TSN is of special importance before applying TSN as a clinical treatment for cancer. The significant difference in the ER status among the three tested cell lines drew our attention, in which the TSN-sensitive cells (U87 and C6) uniquely express ERβ while the TSN-resistant cells (T98G) uniquely express ERα. Recently, a released TCGA (The Cancer Genome Atlas) pilot project ranks ERβ as the top ranking gene for GBM and showed the loss of ERβ during GBM progression [28,29]. Although activation or overexpression of ERβ has been shown to reduce cell proliferation in several cancers, including those of the breast, ovary, prostate, and colon [12,13,30,31], the therapeutic significance of ERβ in GBM remains elusive. Our pharmacological and molecular experiments suggested that TSN-induced inhibition of U87 and C6 cell growth and promotion of apoptosis were correlated with the upregulation of ERβ. We also demonstrated that siRNA-mediated silencing of ERβ abrogated its cytotoxicity. These data are consistent with the general notion that ERβ is a tumor suppressor and that selective ERβ agonists are candidates for anti-cancer drugs [32,33]. According to our study, TSN seems to have no desirable effect on ERα even though ERα and ERβ are structurally similar. We therefore proposed that TSN may have a higher binding affinity to ERβ, which leads to the cytotoxicity in ERβ-positive GBM cells. The absence of endogenous ERβ in T98G cells failed to provide an appropriate target for TSN (e.g., “off-target”) and resulted in the unresponsiveness to this treatment.

The ectopic expression of ERβ in T98G cells, however, failed to restore sensitivity to TSN. Several lines of evidence in colon cancer have highlighted the importance of p53 in ERβ-mediated tumor suppression [26]. Our results also suggested that activation of functional p53 is closely associated with apoptosis induction. We concluded that the cytotoxicity of TSN to GBM cells is a stepwise biological event involving upregulation of ERβ and activation of wild-type p53. Both steps are required for triggering the apoptosis of GBM cells, and defects in either pathway would compromise the anti-cancer effect of TSN. Therefore, TSN would be an alternative therapeutic strategy for patients with GBM expressing endogenous ERβ and functional p53, and these two molecules could serve as biomarkers underlying the responsiveness to TSN. Screening for the ERβ and p53 status is necessary to discriminate GBM patients who may benefit from TSN or other agents that manipulate these pathways.

It is likely that the ERβ- and p53-mediated cytotoxicity of TSN is not only relevant in the settings of GBM. In a broader context, TSN may kill other cancer cells expressing ERβ and p53. Exposure of MCF-7 breast cancer cells (ERβ+/p53 wild type) to TSN leads to profound apoptosis, whereas T47D breast cancer cells (ERβ+/p53 L194F mutated) poorly respond to this treatment (Figure S5). This cell-specific difference in TSN response is likely dependent on the differential expression and function of p53. We proposed that the triple-negative breast cancer cells may not be sensitive to TSN because of their lack of ERβ expression. Future studies in other types of cancer may also correlate the ERβ and p53 status with TSN cytotoxicity.

Collectively, we reported the anti-cancer effect of TSN in GBM cells through the induction of ERβ and functional p53. While TSN in its natural form has relatively low water solubility and bioavailability, preclinical development of TSN derivates such as liposomal TSN, nanoformulations of TSN, and co-encapsulated TSN, should be underway for future studies.

## 4. Materials and Methods

### 4.1. Cell Culture

The human GBM cell lines U87 and T98G were purchased from American Type Culture Collection (ATCC, Manassas, VA, USA), and the rat GBM cell line C6 was purchased from the Chinese Academy of Sciences (Shanghai, China). The cells were cultured in Dulbecco’s modified Eagle’s medium (Life Technologies, Carlsbad, CA, USA) supplemented with 10% FBS (fetal bovine serum) (HyClone, Logan, UT, USA) and penicillin/streptomycin at 37 °C with a 5% CO_2_ atmosphere in a humidified incubator. All of the cell lines were passaged for less than three months, and cell stocks were available in liquid nitrogen.

### 4.2. MTT Assay

U87 and C6 cells were seeded in 96-well plates at a density of 5 × 10^3^ cells per well and treated with TSN (Nanjing Spring & Autumn Biological Engineering Co., Nanjing, China) at the indicated concentrations and time intervals. The content and purity of TSN have been confirmed by chromatograms (Figure S6). A sterile MTT solution (Sigma, St. Louis, MO, USA) was added to each well, and the cells were incubated for an additional 4 h. The medium was then removed, and 150 µL dimethyl sulfoxide (DMSO) was added to dissolve the formazan crystals formed in the viable cells. The plates were read at 570 nm (OD_570_) using a microplate reader (BioRad, Hercules, CA, USA). At least three independent experiments were performed.

### 4.3. Colony Formation Assay

A total of 2.5 × 10^3^ cells were seeded into a six-well plate and allowed to attach overnight. Cells were then treated with 1 nM TSN, and the medium was refreshed every two days. After 10 days of treatment, colonies were fixed and stained with Giemsa solution. Plates were washed with phosphate buffer saline (PBS), air dried, and photographed.

### 4.4. Flow Cytometry Analysis of Apoptosis

Cell apoptosis was analyzed using an Annexin V/PI apoptosis detection kit (BD Biosciences, San Jose, CA, USA) following the instructions of the manufacturer. Briefly, 5 × 10^5^ cells were seeded in a six-cm dish and treated with 10 nM TSN. After 48 h of treatment, the cells were harvested, washed with cold PBS (pH 7.4), centrifuged, and double-stained with annexin V-FITC and PI in binding buffer (10 mM HEPES (pH 7.4), 140 mM NaCl, 2.5 mM CaCl_2_) for 15 min in the dark. The samples were analyzed by flow cytometry.

### 4.5. Western Blotting

Cells were lysed with a lysis buffer (Beyotime, Shanghai, China), and total protein concentration was determined by the Bicinchoninic Acid assay. Equal protein amounts were resolved using sodium dodecyl sulfate-polyacrylamide gel electrophoresis and transferred to 0.45 μm nitrocellulose membranes. After they were blocked with 5% non-fatty milk at room temperature for 60 min, the membranes were incubated with primary antibodies against Bcl-2 (1:1000; Abcam, Cambridge, MA, USA), Bax (1:1000; Abcam), Bak (1:1000; Cell Signaling Technology, Danvers, MA, USA), Bad (1:1000; Cell Signaling Technology), ERα (1:1000; Abcam), ERβ (1:1000; Abcam), p53 (1:1000; Cell Signaling Technology), and β-actin (1:5000; Millipore, Billerica, MA, USA) at 4 °C overnight. The membranes were then washed three times with PBST buffer (PBS and 0.05% Tween 20) and incubated with horseradish peroxidase (HRP)-conjugated secondary antibodies (1:5000; Cell Signaling Technology). Protein bands were then detected by chemiluminescence.

### 4.6. Xenograft Models

To assess the anti-cancer effect of TSN in vivo, we implemented the xenograft mouse model. Ten million U87-Luc cells were suspended in 100 µL Matrigel (BD Biosciences, San Jose, CA, USA) and subcutaneously injected into the bilateral flanks of six-week-old null mice (*n* = 10). When the tumor volume reached approximately 250 mm^3^, the mice were assigned to receive either vehicle or TSN via oral gavage (*n* = 5 in each group). For in vivo bioluminescence imaging, mice were injected with D-Luciferin (Promega, Madison, WI, USA) after 10 days of treatment. The luminescence signal was recorded by using the Xenogen-IVIS Imaging System. Mice were then humanely sacrificed, and the tumors were carefully isolated and processed for histological studies. All mouse experiments were approved by the Animal Ethics Committee of the Fourth Military Medical University (Animal Experimental Ethical Inspection Registration Number: 20160105; Animal Entering Date: 10 March 2016; Animal Ending date: 15 April 2016).

### 4.7. Immunohistochemistry and TUNEL Assay

Tumor samples were fixed in 4% formalin overnight and prepared as paraffin-embedded stocks. Hematoxylin and eosin staining and immunohistochemistry were performed according to standard procedures. The slides were incubated with primary antibodies against Ki67 (1:200, Abcam), Bcl-2 (1:100, Abcam), Bax (1:100, Abcam), and cleaved caspase-3 (1:100, Abcam) followed by treatment with HRP-conjugated secondary antibody (Dako, Glostrup, Denmark). Immunoreactivity was visualized with the Dako EnVision™ Detection kit (Dako). The terminal deoxynucleotidyl transferase-mediated dUTP nick end labeling (TUNEL) assay was performed following the manufacturer’s instructions (Roche, Basel, Switzerland). Briefly, the slides were digested with proteinase K, blocked with H_2_O_2_, and incubated with the terminal deoxynucleotidyl transferase mixture for 60 min. The slides were thoroughly washed, incubated with streptavidin-HRP, and visualized by diaminobenzidine.

### 4.8. siRNAs, Constructs, and Transfection

siRNAs (Small interfering RNAs) against ERβ, p53, or GFP was synthesized by Augct Bio-Tech Co. (Beijing, China) and transfected into GBM cells by Lipofectamine™ 2000 (Invitrogen, Carlsbad, CA, USA). The siRNAs sequences used were as follows: siERβ: 5′-AAUAUCUCUGUGUCAAGGCCA-3′; sip53: 5′-GAGUGCAUUGUGAGGGUUAUU-3′; and siGFP: 5′-CGCUGACCCUGAAGUUCAU-3′. Full-length ERβ and wild-type p53 fragments were polymerase chain reaction (PCR) amplified and inserted via In-Fusion into the pcDNA3.1 vector downstream of a cytomegalovirus promoter. All of the constructs were thoroughly sequenced and transfected into GBM cells. Cells were selected in 1 µg/mL G418 (MP Biomedicals, Santa Ana, CA, USA) for two weeks, and the G418-resistant clones were analyzed for ERβ and p53 protein expression.

### 4.9. Statistical Analysis

The data are presented as the mean ± standard error of at least three replicates. Statistical analysis was performed using analysis of variance followed by Student’s *t*-test to determine the differences among the groups. * *p* < 0.05 was considered to be statistically significant.

## 5. Conclusions

Our study indicates a promising strategy for treating GBM. TSN inhibits GBM cell proliferation and induces apoptosis in vitro and in vivo. The cytotoxicity of TSN in GBM depends on induction of ERβ and functional p53. Screening ERβ and p53 status may help to identify patients who could benefit from TSN treatment.

## Figures and Tables

**Table 1:** The source and genetic background of GBM cells in the present study.

Cell Line	Species	ERα	ERβ	P53
U87	Human	-	+	Wild Type
C6	Rat	-	+	Wild Type
T98G	Human	+	-	Mutated

## Supplementary Materials

Supplementary materials can be found at www.mdpi.com/1422-0067/17/11/1928/s1.

## Abbreviations

ATCC	American Type Culture Collection
DMSO	Dimethyl sulfoxide
ER	Estrogen receptor
FBS	Fetal bovine serum
GBM	Glioblastoma
HRP	Horseradish peroxidase
MAPK	Mitogen-activated protein kinase
MTT	3-(4,5-Dimethyl-2-thiazolyl)-2,5-diphenyl-2-*H*-tetrazolium bromide
PBS	Phosphate buffer saline
PCR	Polymerase chain reaction
PI3K	Phosphatidylinositol 3 kinase
siRNAs	Small interfering RNA
TCGA	The Cancer Genome Atlas
TSN	Toosendaninz
TUNEL	Minal deoxynucleotidyl transferase dUTP-mediated nicked end labeling
