# Supplementary material for: Toosendanin Exerts an Anti-Cancer Effect in Glioblastoma by Inducing Estrogen Receptor β- and p53-Mediated Apoptosis
Source: Int J Mol Sci. 2016 Nov 18;17(11):1928. doi: 10.3390/ijms17111928 (PMC5133924; doi:10.3390/ijms17111928)
Supplement: Supplementary file 1 [file ijms-17-01928-s001.pdf]

Supplementary Material: Toosendanin Exerts an Anti-Cancer Effect in Glioblastoma by Inducing Estrogen Receptor β- and p53-Mediated Apoptosis

Liang Cao, Dingding Qu, Huan Wang, Sha Zhang, Chenming Jia, Zixuan Shi, Zongren Wang, Jian Zhang and Jing Ma

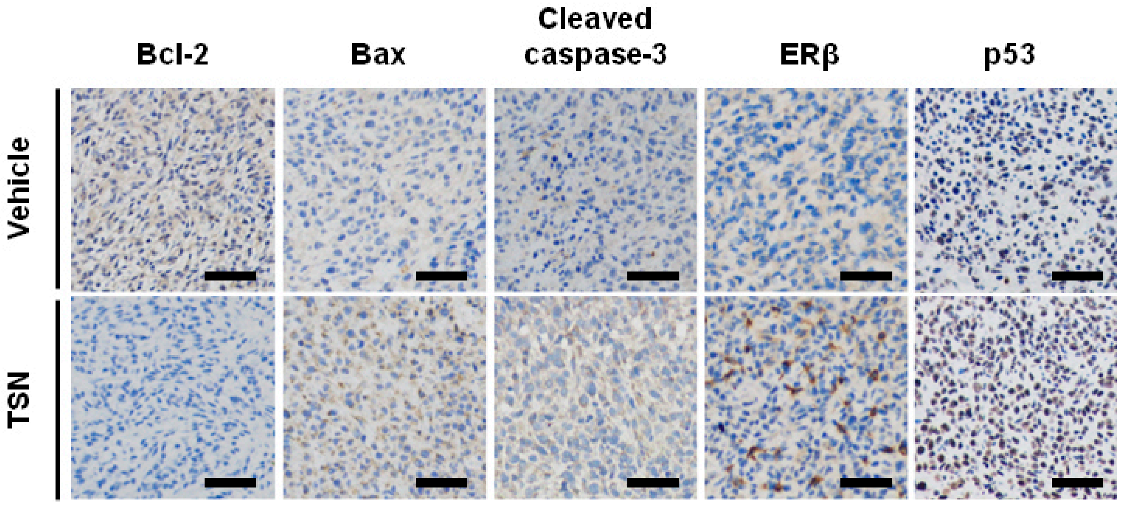

Figure S1. Effect of TSN (toosendanin) on U87 cells apoptosis and ERβ (estrogen receptor β) and p53 induction in vivo. The U87 xenograft tumors were fixed in 4% formalin, cut into 5 μm slides, and evaluated for Bcl-2, Bax, cleaved caspase-3, ERβ, and p53 protein expression by immunohistochemistry (Scale bar = 100 μm).

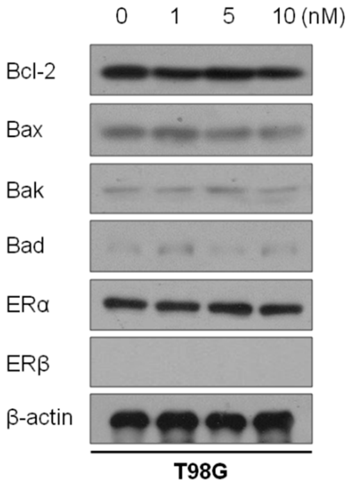

Figure S2. Western blot analysis of apoptosis related proteins and ERs proteins expression in TSN-treated T98G cells.

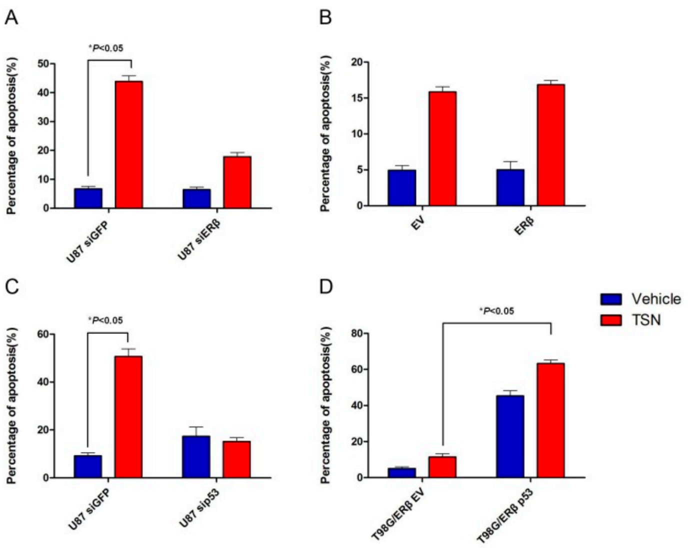

Figure S3. Flow chart of apoptosis related to Figure 4C (**A**), Figure 4E (**B**), Figure 5B (**C**) and Figure 5D (**D**).

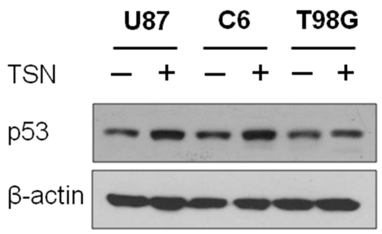

Figure *S4*. Effect of TSN on p53 expression. U87, C6, and T98G GBM cells were treated with 10 nM TSN for 48 h and examined for p53 protein level.

Cell Line	Species	ERα	ERβ	P53
MCF-7	Human	+	+	Wild Type
T47D	Human	+	+	Mutated

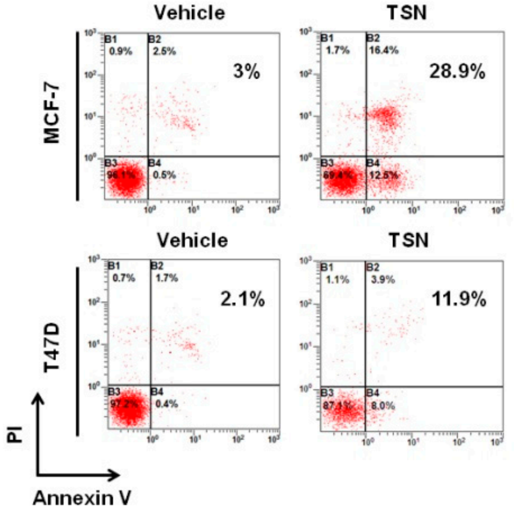

Figure *S5*. Effect of TSN on apoptosis in breast cancer MCF-7 and T47D cells. MCF-7 and T47D cells were treated with 10 nM TSN. Representative flow cytometry images were shown.

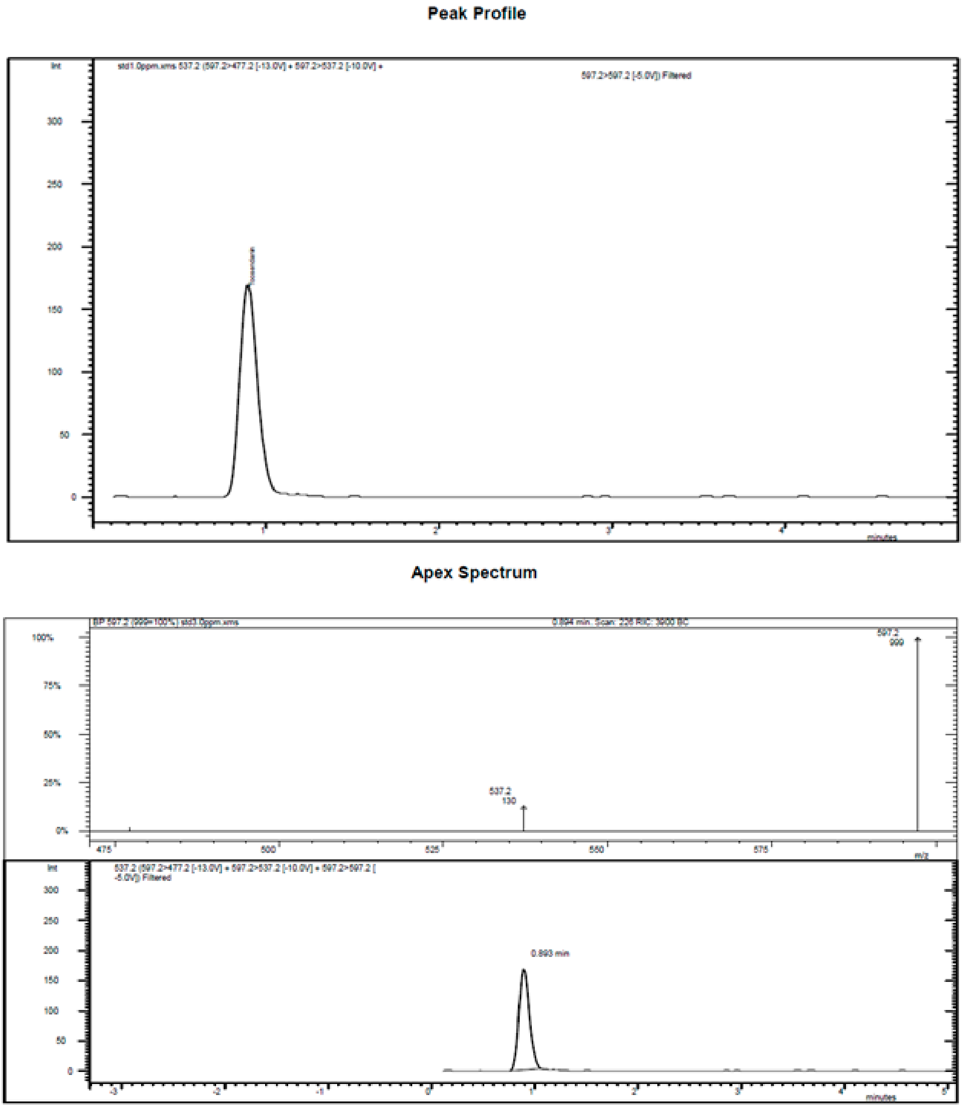

Figure S6. Chromatograms analysis of TSN purity.
